# Clinical characteristic and intraoperative findings of uterine perforation patients in using of intrauterine devices (IUDs)

**DOI:** 10.1186/s10397-017-1032-2

**Published:** 2018-01-16

**Authors:** Xin Sun, Min Xue, Xinliang Deng, Yun Lin, Ying Tan, Xueli Wei

**Affiliations:** Department of Obstetrics and Gynecology, The 3rd Xiangya Hospital of Central South University, 138 Tongzipo Rd, Changsha, Hunan 410013 China

**Keywords:** IUD, Uterine perforation, Hysteroscopy, Laparoscopy

## Abstract

**Background:**

Intrauterine devices (IUDs) are the most popular form of contraception used worldwide; however, IUD is not risk-free. IUD migrations, especially uterine perforations, were frequently occurred in patients. The aim of this study was to investigate the clinical characteristics and intraoperative findings in patients with migrated IUDs.

**Results:**

29 cases of uterine perforation associated with migrated IUDs and 69 control patients were followed between January 2008 to March 2015. Patients who used IUDs within first 6 months from the last delivery experienced a characteristically high rate of the perforation of the uterine wall. A significantly larger number of IUD insertion associated with uterine perforation were performed in rural hospitals or operated at a lower level health care system. There was no clear difference in the age and presented symptoms in patients between two groups. Majority of contraceptive intrauterine devices was the copper-releasing IUDs. Furthermore, patients who used V-shaped IUD showed significantly higher incidence of pelvic adhesions when compared with the users of O-shaped IUDs.

**Conclusions:**

Unique clinical characteristics of IUD migration were identified in patients with uterine perforation. Hysteroscopy and/or laparoscopy were the effective approaches to remove the migrated IUDs. Improving operating skills is required at the lower level of health care system.

## Background

Intrauterine devices (IUDs) are the most popular form of contraception used by millions of women worldwide, particularly in developing countries [1]. Currently, two major types of IUDs are used, the copper-releasing intrauterine device (IUD) and the levonorgestrel-releasing intrauterine system (LNG-IUS) [2, 3]. However, IUDs are not risk-free, and lots of complications have been reported, including expulsion, malpositioning, and uterine perforation [4]. Among them, the majority were missing the IUDs, and most of the missing IUDs were found in the uterus [2]; however, the device would also pass through the uterine serosa causing uterine perforation. Uterine perforation is rare, but it may cause serious problems, including pain, abnormal bleeding, bowel or bladder perforation, and fistula formation, when IUDs migrate into the pelvic peritoneal space invading the adjacent organs. Thus far, the invasion of omentum, pouch of Douglas, the serosa of the ileum, the bladder, and the rectum has been reported in patients with uterine perforation [3, 5–8]. The rate of uterine perforation was 0.3–2.6 in every 1000 users of copper IUD insertion, and the respective rate for LNG-IUS insertion was 0.3–2.2 [9–15]. Uterine perforation can be primary (at the time of insertion) or secondary (4 weeks or more after the insertion took place). Lots of risk factors were proposed for uterine perforation, including immediate post-partum period [10, 16], breastfeeding [14, 17], extremes of uterine posture [18], intrauterine device shape not fitting well with uterine cavity [19], inexperience of the inserter, and inappropriate technique during the IUD insertion [15, 16, 20].

Specifically, the use of intrauterine devices has been changing in parallel with social-economic development of China. In the 1980s, 90% of all IUDs were stainless steel rings; they were cheaper than copper T-shaped IUDs but were also less effective and had a greater rate of expulsion [1]. In the 1990s, the use switched to T-shaped copper IUDs, although a large population of women was still using the steel rings [21]. Today, there are dozens of different types of IUDs available on the market that clearly mirror their clinical application [22–24]. Most of the IUDs used in China are manufactured in China, and the majority is the copper-releasing IUDs, including all types of T-shaped and V-shaped copper-releasing IUDs. Recently, some overseas products, as LNG-IUS [25] and GyneFix [26], have also been introduced to China.

Although lots of factors have been proposed, the precise cause of uterine perforation is not well understood [27]. The risk factors associated with use of IUDs by Chinese women were not reported. In this retrospective study, we attempted to evaluate all potential risk factors for uterine perforation associated with the use of IUDs by Chinese women and provide suggestions possibly useful for the management of IUD migrations.

## Methods

### Patients enrolled and surgery management

Patients admitted to the 3rd Xiangya Hospital of Central South University (CSU) at Hunan, China, from January 2008 to March 2015 were reviewed for uterine perforation. Uterine abnormalities were evaluated by by B-scan ultrasonography (B-scan), X-rays, or computed tomography (CT). Two groups of patients with (1) uterine perforation and (2) non-uterine perforation were enrolled into the study. In the event of uterine perforation, the IUD devices passed through the uterus and caused uterine injury; 29 cases were included in this study. In the patients without uterine perforation (“non-uterine perforation”), there was a displacement of IUD, but the device was still located in the uterus; 69 cases were included in the study. Patients who had preexisting IUDs but could not find the missing IUDs, which might be due to expulsion, were excluded from the study. This study was approved by the Review Board and Ethics Committee of the 3rd Xiangya Hospital of Central South University (# 2008-S010). All patients signed a statement of consent to participate under the “ethics, consent, and permissions” heading and another informed consent to the publication of collected data.

In patients with non-uterine perforation, the IUDs were malpositioned in the uterine or embedded in the uterine myometrium but did not cause perforation of the organ. All these patients underwent hysteroscopy as initial step in removing the dislocated IUDs. Patients with uterine perforation had partially or completely perforated organ throughout the uterine serosa. In these patients, laparoscopy and/or hysteroscopy or cystoscopy were applied to remove the migrated IUDs. Since one patient showed perforated ileum; laparotomy was performed to repair the intestine after the removal of the IUD.

### Patient information and intraoperative findings

Patient information, including age, medical history, history of previous pregnancy outcome, symptoms encountered by the patient, the time of IUD insertion after the last delivery, the time from the IUD insertion to diagnosis, and health facility where IUD insertion was conducted, was collected when the patients came to the hospital.

The intraoperative findings were recorded during the surgical operations, which include type and location of IUD, site of uterine perforation, surgical management, and pelvic adhesion. These and other associated complications were also recorded during hysteroscopy and/or laparoscopy, cystoscopy, and laparotomy.

### Statistical analysis

Data analysis was performed using GraphPad Prism ver. 6 (GraphPad Software, La Jolla, CA). A nonparametric, the Mann-Whitney *U* test was used to assess the differences in parameters between the uterine perforation and non-uterine perforation groups, while Fisher’s exact test was applied in the evaluation of the incidence of these two groups.

## Results

### Multiple factors contributed to uterine perforation occurrence

The patients’ characteristics were summarized in Table 1. The average age of all patients was 37, and there was no significant difference between the groups of patients (Mann-Whitney test, *p* > 0.05). The median age was 35 (range 22–60) for uterine perforation and 38 (range 20 to 71) for non-uterine perforation groups (Table 1).

**Table 1:** Characterization of 98 patients with uterine perforation and without uterine perforation in Chinese women of IUD insertion

Characteristic	Uterine perforation (*n* = 29)	Non-uterine perforation (*n* = 69)
Age (years)		
Median (range)	35 (22–60)	38 (20–71)
Symptoms		
Asymptomatic	10 (34.5)	32 (46.4)
Pain (pelvic and/or abdominal)	10 (34.5)	15 (21.7)
Menstrual disorders	3 (10.3)	12 (17.4)
Unintended pregnancy	5 (17.2)	4 (5.8)
Missing strings	4 (13.8)	4 (5.8)
Vaginal bleeding	0 (0)	3 (4.3)
History of previously pregnancy outcome		
Cesarean section	7 (24.1)	27 (39.1)
Vaginal birth	22 (75.9)	42 (60.9)
Diagnosis of IUD migration		
B-scan ultrasonography (B-scan) only	13 (44.8)	52 (75.4)
X-radiation (X-rays) only	3 (10.3)	1 (1.4)
B-scan + X-rays	6 (20.7)*	3 (4.3)
Others	7 (24.1)	13 (18.8)
IUD insertion after last delivery (months)		
Median (range)	16 (3–60)	24 (3–120)
≤ 6	15 (51.7)*	17 (24.6)
> 6 to ≤ 12	4 (13.8)	15 (21.7)
≥ 12	8 (27.6)	20 (29.0)
Not postpartum	2 (6.9)	17 (24.6)
Time from insertion to diagnosis (months)		
Median (range)	114 (1–408)	93 (1–480)
≤ 12	8 (27.6)	13 (18.8)
> 12 to ≤ 60	6 (20.7)	27 (39.1)
> 60 to ≤ 120	6 (20.6)	15 (21.7)
≥ 120	9 (31.0)	14 (20.3)
Institution for operation of IUD inserting		
Rural hospital or lower level	26 (89.7)*	48 (69.6)
Urban hospital	3 (10.3)	21 (30.4)
Type of IUD		
T-shaped copper, made in China	10 (34.5)	20 (29.0)
V-shaped copper, made in China	13 (44.8)	31 (44.9)
O-shaped, made in China	4 (13.8)	10 (14.5)
GyneFix, made in Belgium	1 (3.4)	3 (4.3)
Others	1 (3.4)	5 (7.2)

Non-uterine perforation was subjected to IUD malposition without uterine perforation. The data are presented as *n* (%) unless stated otherwise

*p < 0.05, analyzed by Fisher’s exact test

In this study, we found 10 patients (34.5%) with the uterine perforation showing no signs or symptoms of a disease at hospital arrival screens. Thirty-two patients (46.4%) in the non-uterine perforation group did not have symptoms, either. While a large number of patients were encountered with different symptoms, most of them were presented as pelvic and/or abdominal pain. Ten patients (34.5%) in the uterine perforation group had pain, and 15 (21.7%) of those with pain were observed in the non-uterine perforation group. Pain prevalence was followed by menstrual disorders affecting 10.3% of patients in the uterine perforation and 17.4% of them in the group of non-uterine perforation.

Pregnancy occurred in 17.2% in the uterine perforation group and 5.8% of cases in the non-uterine perforation. About 13.8% of patients with uterine perforation were associated with IUD missing strings and 5.8% of those with non-uterine perforation. Three cases of vaginal bleeding were observed, all of them in the group of non-uterine perforation (Table 1).

The time interval between IUD insertion and the time of last delivery was shorter in the uterine perforation group when compared with the group of non-uterine perforation. A half of the patients (*n* = 15, 51.7%) with the uterine perforation inserted the device within 6 months after delivery, which is significantly higher than in the patients of the no uterine perforation group (*n* = 17, 24.6%;Table 1, Fisher’s exact test, * = *p* < 0.05). The time passed from the insertion of IUD to diagnosis of uterine perforation (duration of IUD placement) tended to be longer (14 months in average) when compared with 93 months for the conditions of non-uterine perforation (Table 1).

There are three major types of IUDs that were typically used: T- and V-shaped copper-releasing IUDs and O-shaped IUDs (O-shaped IUDs referred to as stainless steel single or double rings). Our findings showed that uterine perforation cannot be associated with a particular type of IUD (Table 1). However, 89.7% (*n* = 26) of patients with uterine perforations inserted IUDs in rural hospitals or at lower level health care systems, in comparison with 69.6% (*n* = 48) of those with no uterine perforation treated in the same setting Table 1).

### Intraoperative findings were associated with uterine perforation

If the IUDs were going through the uterine serosa, or entirely outside the uterus, invading the organs in the pelvic abdominal cavity would make it difficult to remove devices. This invokes an increased risk for severe complications.

There was not a clear pattern of the organs affected by displaced and protruding IUDs through the uterine. Myometrium appeared on the top of the scattered pattern of organ injury (*n* = 6, 20.7%) and greater omentum (*n* = 6, 20.7%), followed by the sigmoid colon (*n* = 5, 17.2%) (Fig. 2), left sacrouterine ligament (*n* = 3, 10.3%), bladder (*n* = 3, 10.3%), pouch of Douglas (*n* = 2, 6.9%), and rectal serosa (*n* = 2, 6.9%). One patient (3.4%) had injured uterus isthmus and affected ileum was observed in the other one (3.4%; Table 2).

**Table 2:** Location and type of IUDs identified in uterine perforation

Location	Occurrence of patients	Type of IUDs				
		T-shaped copper	V-shaped copper	O-shaped	GyneFix	Others
Myometrium	6/29 (20.7)	1/10 (10.0)	4/13 (30.8)	1/4 (25.0)	0/1 (−)	0/1 (−)
Greater omentum	6/29 (20.7)	1/10 (10.0)	4/13 (30.8)	0/4 (−)	1/1 (100)	0/1 (−)
Sigmoid colon	5/29 (17.2)	2/10 (20.0)	3/13 (23.1)	0/4 (−)	0/1 (−)	0/1 (−)
Left sacrouterine ligament	3/29 (10.3)	0/10 (−)	0/13 (−)	3/4 (75.0)	0/1 (−)	0/1 (−)
Bladder	3/29 (10.3)	2/10 (20.0)	1/13 (7.7)	0/4 (−)	0/1 (−)	0/1 (−)
Pouch of Douglas	2/29 (6.9)	2/10 (20.0)	0/13 (−)	0/4 (−)	0/1 (−)	0/1 (−)
Serosa of rectum	2/29 (6.9)	1/10 (10.0)	1/13 (7.7)	0/4 (−)	0/1 (−)	0/1 (−)
Isthmus	1/29 (3.4)	1/10 (10.0)	0/13 (−)	0/4 (−)	0/1 (−)	0/1 (−)
Ileum	1/29 (3.4)	0/10 (−)	0/13 (−)	0/4 (−)	0/1 (−)	1/1 (100)

The data are presented as *n* (%) unless stated otherwise

**Fig. 1:**
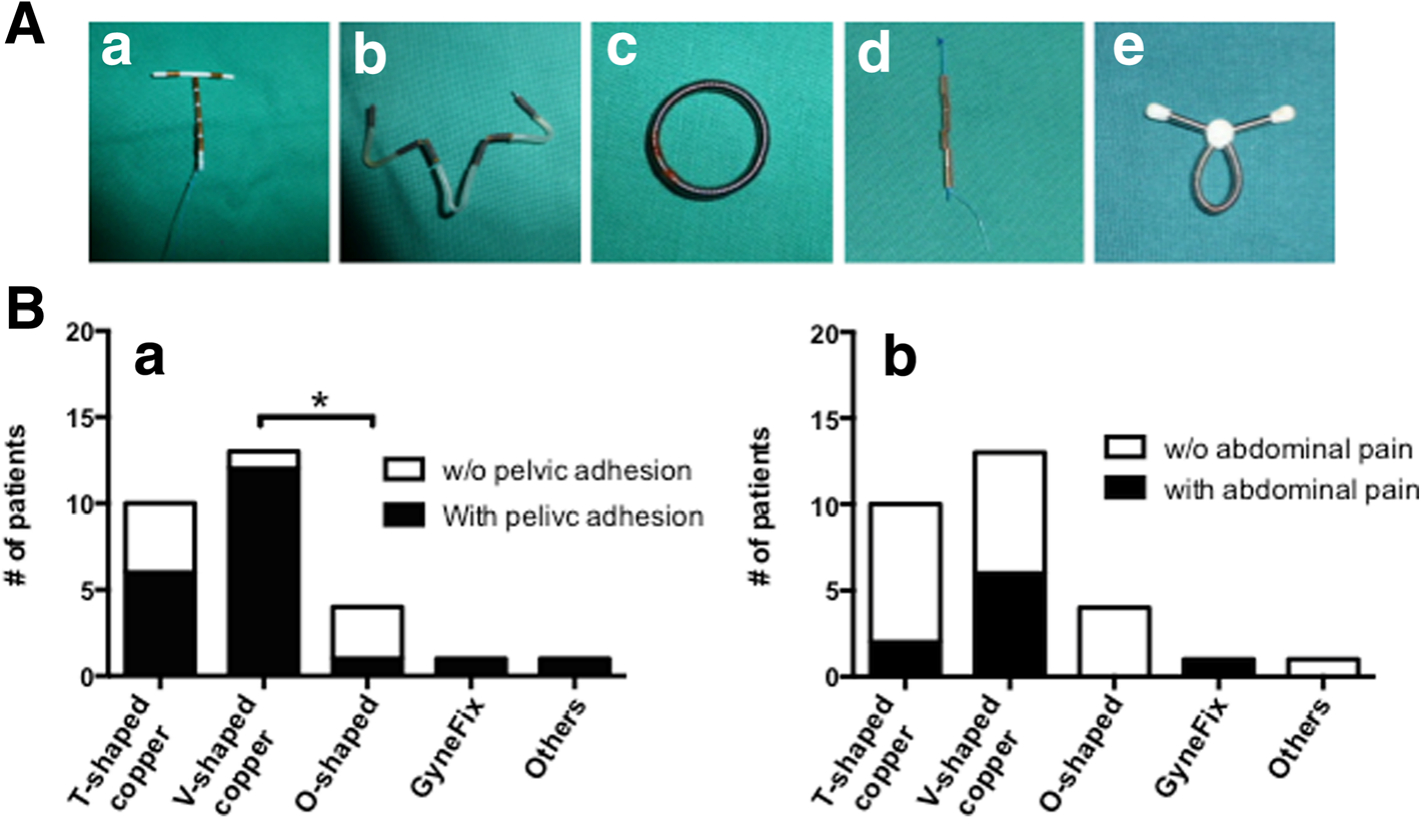
IUDs and complications were associated with uterine perforation. **A** Different types of IUDs were identified in the patients with uterine perforation, including T-shaped copper (**a**), V-shaped copper (**b**), O-shaped (**c**), GyneFix (**d**), and other type (**e**) of IUDs. **B** Pelvic adhesion and abdominal pain were associated with uterine perforation. Five major types of IUDs were correlated with the intraoperative finding of pelvic adhesion (**a**) and clinical symptom of abdominal pain (**b**). Please note that significantly higher ratio of pelvic adhesions were observed in V-shaped copper IUD patients of 12/13 (92.3%) compared with O-shaped IUD patients of 3/4 (75%) with uterine perforation (Fisher’s exact test, *p < 0.05), while the abdominal pains were no different among all the types of IUDs used with uterine perforation. Note, w/o means without

We identified five types of IUDs that were implanted (Fig. 1A (a–e). Most of them are T-shaped copper-containing device (*n* = 10, 34.5%) and V-shaped copper-containing device (*n* = 13, 44.8%), which showed a random invasion of the pelvic peritoneum. The O-shaped IUDs predominantly invaded the left sacrouterine ligament (3/4, 75%; Table 2).

Uterine perforation frequently causes pelvic adhesion as reported before [6], but it is still unknown whether any typical type of IUD that is made in China is associated with pelvic adhesion. In our study, we found that V-shaped copper-releasing IUDs (12/13, 92.3%) induced significantly higher rate of pelvic adhesion when compared with the O-shaped IUDs (1/4, 25%) (Fig. 1B (a), Fisher’s exact test, * = *p* < 0.05). The pain induction could not be related to any particular device evaluated in this study (Fig. 1B (b)).

### Laparoscopy and/or hysteroscopy were applied to remove the migrated IUDs

**Fig. 2:**
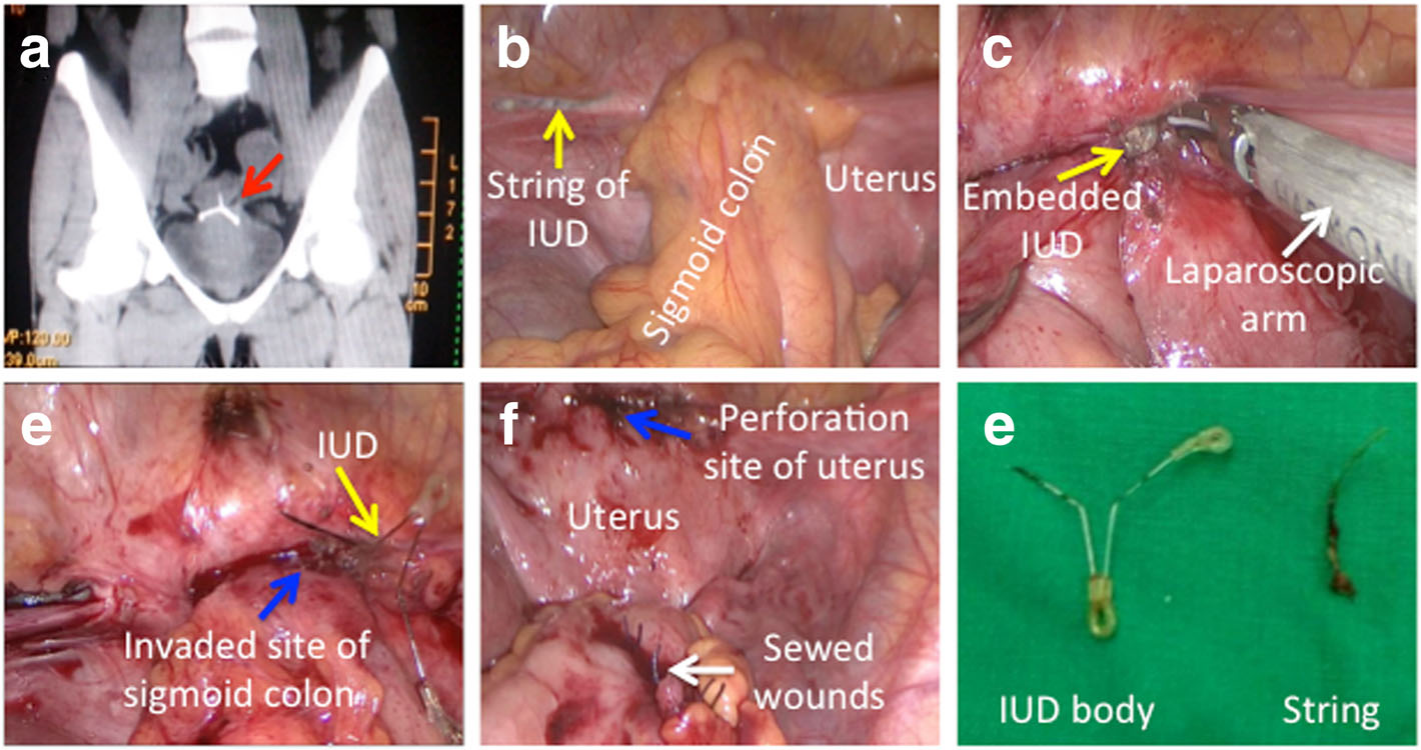
Migrated IUD was removed by laparoscopy in a typical case of uterine perforation. **a** A 36-year-old patient was visiting the 3rd Xiangya Hospital for checking of hysteromyoma in January 2015, and this patient was experienced with two types of IUD insertions, and because of that, she assumed that the first IUD was expelled. However, after she took out the second IUD, another IUD was monitored unexpectedly by computed tomography (CT), indicating that the first IUD had undergone uterine perforation. Note that the red arrow showed the IUD. **b**–**f** The laparoscopy was applied for the removal of the migrated IUD. It was shown that the IUD completely perforated through the uterine serosa and invaded into the sigmoid colon and, finally, the IUD was removed and the wounds were sewn under laparoscopy. Note that the migrated IUD was shown in yellow arrows, and the perforation site at the uterus and the invaded site of the sigmoid colon were shown in blue arrows. **e** The IUD was identified as a V-shaped copper IUD, and the string was already separated from the IUD body

Once the patients were diagnosed with uterine perforation and the device’s location was identified, they underwent surgical removal of migrated IUDs. Five patients (17.2%) out of 29 underwent laparoscopy for removal of the IUDs, while 22 patients (75.9%) underwent hysteroscopy along with laparoscopy (Table 3 and Fig. 2). Since one patient experienced ileum injury, it underwent laparotomy for exploration, IUD removal, and the wound repair. In the other patient, the IUD migrated into the bladder, and the device was removed by cystoscopy (Table 3). All the patients showed signs of healthy recovery during hospitalization and were discharged within 1 week.

**Table 3:** Surgical approaches for uterine perforation cases

Approaches	Treatment of patients, *n* (%)
Laparoscopy	22/29 (75.9)
Hysteroscopy + laparoscopy	5/29 (17.2)
Laparotomy	1/29 (3.4)
Cystoscopy	1/29 (3.4)

## Discussion

Uterine perforation is a serious complication associated with IUD use. The precise mechanism of induction of uterine perforation is not well understood. A lot of risk factors were proposed for the induction of uterine perforation [10, 14, 15, 19]. In this study, we found that the time of IUD insertion after the last delivery is shorter in the uterine perforation group when compared with the subjects without uterine perforation. A sharp increase of the uterine perforation rate occurred in the subjects with IUD inserted up to 6 months after delivery. The incidence of uterine perforation within the first 6 months was significantly higher when compared with the subjects treated under the same conditions but without uterine perforation (Table 1, 51.7 vs. 24.6%, *p* < 0.05). At this stage, most patients were still in the breastfeeding period, and all 15 subjects in the lactation period were diagnosed with uterine perforation. This finding is consistent with previous reports that women in the lactation period are at a high risk of uterine perforation [14, 17]. In addition, most of the patients with the uterine perforation had received an IUD at rural hospitals or a lower level of health care systems. Typically, the experience and technique of an obstetrician-gynecologist in rural hospitals or lower level health care systems are poorer when compared to those in urban hospitals in China. Many obstetrician-gynecologists in rural hospital or a lower level of health care systems are lacking an efficient training on handling of IUD insertion, which may directly cause perforation at the insertion time. As it was reported before, IUD migrations at the time of insertion may directly contribute to uterine perforation [14, 17, 28].

The IUD use in China has a long history, and it can be found as earlier as 1979 [1]; however, the IUD devices were still not well managed. In the early years, most IUDs were steel rings (designated as O-shaped in this study) due to its low cost, and the devices were provided free of charge in some hospitals [1, 22]. Later, the copper-releasing IUDs replaced the steel rings as many problems were encountered by using the rings [29].Nowadays, copper-releasing IUDs have become the prevailing devices in clinical application in China [22, 24]. Recently, the levonorgestrel intrauterine system (LNG-IUS) along with other optimized IUDs were also provided to Chinese women [25]. In this study, the majority of devices that induced uterine perforation were copper-releasing IUDs with T-shaped copper (*n* = 10) followed by V-shaped copper IUDs (*n* = 13) and four of those with O-shaped IUDs (Table 1). A larger number of patients is required to assess the risk factors associated with different types of IUDs, including the LNG-IUS that are used in China.

Pelvic adhesions as a consequence of uterine perforation also frequently occurred in the patients, but the mechanism is still unknown; it may due to different actions of the devices [3]. In our study, we found that 12 out of the 13 patients with V-shaped contraceptive intrauterine devices had pelvic adhesions (Fig. 1B). This is significantly higher when compared with the patients that used O-shaped IUDs. The reason(s) for the higher rate of pelvic adhesions in patients who used V-shaped IUD is unknown at present. This issue requires a thorough investigation.

If the IUDs were missing, a method of searching for the strings or IUDs in the uterine cavity is sweeping by using uterine forceps. However, there are many IUDs used by Chinese women that do not have any string [23] (Fig. 1). The exploration success rate highly varies and is dependent on the experience and applied technique by the obstetrician-gynecologist who removes the IUDs using forceps or hooked instruments [30]. Under these aggravating circumstances, hysteroscopy and/or laparoscopy are suggested procedures, which secure a very high success rate [6–8, 31]. If the IUDs migrated into the pelvic peritoneal cavity, the procedures become dubious. Currently, the WHO recommends that all misplaced IUDs should be surgically removed once they are identified [32]. Some researches, however, suggest that if the migrated IUDs do not cause serious complications or significant symptoms, their removal is not necessary [33, 34]. In our study, all displaced IUDs associated with uterine perforation were successfully removed using laparoscopy and/or hysteroscopy that was accompanied with significant complications. Laparoscopy and/or hysteroscopy provide a high success rate in the removal of migrated IUDs in the patients with uterine perforation.

## Conclusions

Potential risk factors associated with IUD use for the uterine perforation in Chinese population of patients are identified in a retrospective observational study. Hysteroscopy and/or laparoscopy are effective approaches in removing of displaced IUDs. A tailored training program planned to meet patients’ needs in rural areas and at a lower level of health care system may be justified.
